# Glossectomy in the severe maxillofacial vascular malformation with jaw deformity: a rare case report

**DOI:** 10.1186/s40902-015-0043-z

**Published:** 2015-11-14

**Authors:** Min-Hyeog Park, Chul-Man Kim, Dong-Young Chung, Jun-Young Paeng

**Affiliations:** 1 Department of Oral and Maxillofacial Surgery, School of Dentistry, Kyungpook National University, Daegu, Republic of Korea; 2 Department of Oral and Maxillofacial Surgery, Kyungpook National University Hospital, 2175 Dalgubeoldae-ro, Daegu, 700-705 Korea

**Keywords:** Macroglossia, Glossectomy, Venous malformation

## Abstract

In the field of oral-maxillofacial surgery, vascular malformations present in various forms. Abnormalities in the size of the tongue by vascular malformations can cause mandibular prognathism and skeletal deformity. The risk in surgical treatment for patients with vascular malformation is high, due to bleeding from vascular lesions. We report a rare case of macroglossia that was treated by partial glossectomy, resulting in an improvement in the swallowing and mastication functions in the patient. A 25-year-old male patient with severe open-bite and mandibular prognathism presented to our department for the management of macroglossia. The patient had a difficulty in food intake because of the large tongue. Orthognathic surgery was not indicated because the patient had severe jaw bone destruction and alveolar bone resorption. Therefore, the patient underwent partial glossectomy under general anesthesia. There was severe hemorrhaging during the surgery, but the bleeding was controlled by local procedures.

## Background

Venous malformations are anomalies of veins or lymph vessels or both veins and lymph vessels. They are present at birth and manifest at different ages. Venous malformations in the tongue cause significant clinical problems such as swallowing difficulties and speech and airway obstruction. The incidence of vascular malformation has been reported in approximately 7 % of all benign tumors, the majority of which develop in the head and neck region. However, when localized on the tongue, in most cases, these lesions can cause clinical problems such as active bleeding and airway obstruction from the mouth [1–3].

The current methods of treatment include electrocoagulation, cryotherapy, sclerotherapy, surgical excision, or combinations of these treatments. The treatment of superficial, localized venous malformation is relatively simple and effective. However, the treatment of deep, extensive venous malformation remains difficult and presents various complications. For complicated cases, the results achieved with a single treatment method are not satisfactory. Therefore, several approaches are required to achieve acceptable results. In most cases, a surgical excision is considered as the first choice to improve the function and appearance. However, for large lesions, partial glossectomy can be considered after sclerotherapy to improve swallowing, chewing, and speech. During the surgery, care should be taken to regulate the hemorrhage and the airway [4–6].

We report a case of surgical treatment using partial glossectomy in a patient who had venous malformation of the tongue.

## Case presentation

**Fig. 1:**
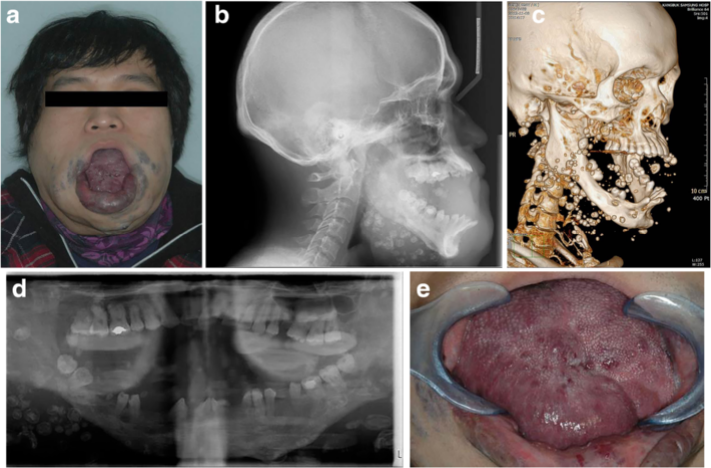
Preoperative patient information. **a** The tongue is interpositioned between the teeth, interfering with chewing. **b** The patient shows severe mandible prognathism, with anterior open-bite. **c** 3-D facial computed tomography view reveals the calcification to be away from the mandible body. **d** Panoramic image shows phleboliths around the mandible body. **e** Clinical photograph shows the macroglossia

**Fig. 2:**
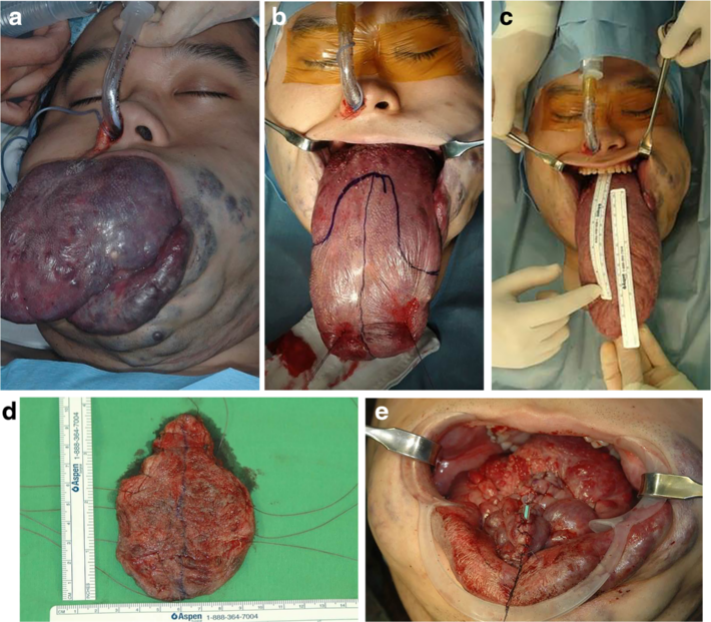
Clinical photos of the surgery. **a** The tongue is protruding between the teeth, under general anesthesia. **b** Glossectomy design. **c** The tongue is taken out of the mouth. **d** Resected tongue measures 7 cm × 10 cm × 4 cm. **e** After glossectomy, a drain is inserted in the anterior part of the tongue to prevent edema

**Fig. 3:**
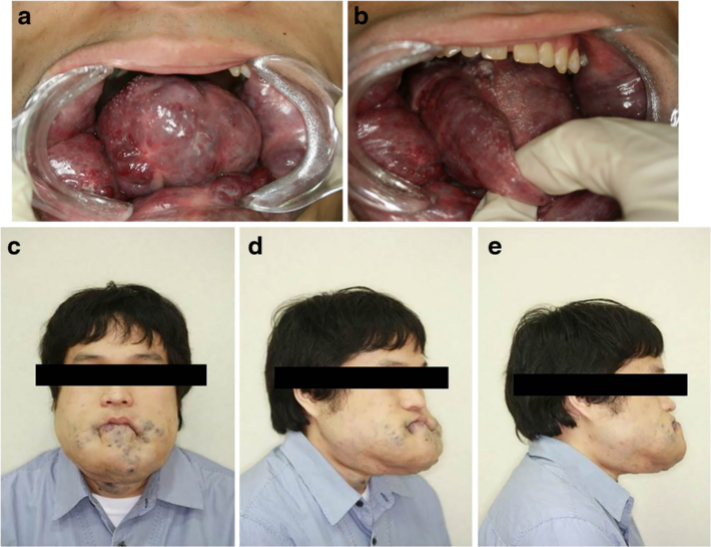
Postoperative patient information. **a**, **b** Complaints of difficulty in breathing, swallowing, and lip incompetence are improved with reduced tongue volume. **c**–**e** Clinical photograph shows the improvement in the open-bite and the lip incompetence

A 25-year-old man with a medical history of macroglossia was referred to our department for the management of the condition on October 25, 2011. The tongue was interpositioned between the teeth, interfering with chewing. He had discomfort in swallowing, chewing, and speech because the vascular mass accounted for most of the tongue. He had been treated previously at a vascular surgery clinic. Surgical resection was performed under general anesthesia during the plastic surgery on January 5, 1996. An excisional biopsy was performed, and the diagnosis of a venous malformation was made. Between 1997 and 2005, ethanol sclerotherapy was performed over ten times. However, the effect of sclerotherapy was unsatisfactory, and the patient continued to experience discomfort in eating because of the large tongue. He exhibited a severe open-bite and mandible prognathism due to the large tongue, at his first visit to our department (Fig. 1a, b, e). In addition, a large amount of phleboliths were observed scattered around the mandible (Fig. 1c, d). Cervical magnetic resonance imaging (MRI) revealed lesions on the mouth floor, the glottis, and the supraglottic area, in addition to the entire tongue. There was no significant interval change in the massive venous malformation in the face and neck since August 11, 2005. Partial glossectomy was performed using the keyhole technique, under general anesthesia, on December 29, 2011 (Fig. 2a, b, c). The incision was performed with a number 15 blade and by electrosurgical coagulation. During the surgery, the excessive tongue mass was removed. The specimen measured 7 cm × 10 cm × 4 cm in size (Fig. 2d). The remaining tongue was sutured with 5-0 Vicryl^®^ sutures, using a half-circle cutting needle. A Penrose drain was inserted in the anterior part of the tongue to prevent edema (Fig. 2e). The endotracheal intubation was retained for 1 week to prevent airway obstruction due to the swelling of the tongue. After the operation, the patient showed mild exudation from the dead space necrotic tissue. Two weeks postoperatively, the patient was discharged without any serious complications. During the follow-up, the patient complained of an impaired sense of taste and a mild, sharp pain in the tongue without any complications of the surgical wound. We prescribed gabapentin (300 mg/day) and cetamadol (975 mg/112.5 mg/day) for the pain, under the impression that it was a neuropathic pain. These medications improved the patient’s pain. Although he experienced some taste impairment, he showed an improvement in swallowing and chewing. There was no recurrence of the lesions on the tongue in a follow-up, 9 months postoperatively (Fig. 3a–e).

### Discussion

Venous malformations are common vascular malformations, presenting at any location, including the head and neck region. They are present at birth, expand slowly during childhood, and often enlarge during trauma, puberty, and pregnancy, due to the hormonal changes occurring during these periods. They are composed of an abnormal collection of veins, which are thin-walled, sponge-like channels of variable size, lacking in smooth muscle. In general, they are a bluish compressible mass and tend to slowly expand with time [7, 8]. For example, a venous infiltration of the tongue results in macroglossia, which presents a risk of swelling or bleeding and may impair swallowing and eating. The tongue plays an important role in swallowing, speech, and breathing, as well as in occlusion and skeletal growth. Therefore, a tongue anomaly may cause a malocclusion and result in changes in skeletal growth, such as open-bite deformity and mandibular prognathism [9, 10].

Venous malformations can be managed by observation, irradiation, electrocoagulation, cryotherapy, low-dose aspirin, sclerotherapy, surgical excision, or combinations of these treatments [1, 11, 12]. The treatment of venous malformation is based on the anatomic regions of the body, the type of tissue, complications such as bleeding, and the functional factors. Therefore, Doppler ultrasonography, magnetic resonance imaging, and direct injection venography may be required to confirm the diagnosis and provide useful information [3]. Sclerotherapy is commonly the preferred treatment method for venous malformations, with surgery performing an adjunctive role. It is a good alternative for venous malformation, given that surgical resection could lead to considerable functional impairment [13]. There are various agents for sclerotherapy, for example, OK-432, ethanol, bleomycin, doxycycline, sodium tetradecyl sulfate, and hypertonic saline, alone or in combination. The potential complications of sclerotherapy include skin and mucosal injuries, swelling, infection, transient nerve palsy, hemoglobinuria, blood loss, and anaphylaxis [14, 15]. Absolute sclerotherapy is recommended alone or with surgery. In our case, surgery was indicated because repetitive sclerotherapy had been ineffective due to the extent of the lesion. Surgery is imperative for extensive lesions.

If airway obstruction is suspected, the treatment should be started even if the lesion is large [16]. Surgical excision results in excellent outcomes only for localized and accessible lesions. Even though surgical excision is the most effective treatment, an excessive excision brings about motor dysfunction, massive bleeding, cosmetic problems, and nerve damage in patients with widespread lesions because of the complicated anatomy of the head and neck region [3, 17]. Our patient had had sclerotherapy several times before surgery, without any improvement. The most clinically concerning aspect to plan a glossectomy was the postoperative airway management [18]. Because the lesion involved the neck around the trachea, a tracheostomy could have caused uncontrollable bleeding through the surgical site. Postoperative airway management was performed by maintaining postoperative nasotracheal intubation for several days. However, the postoperative swelling of the tongue was relatively extended, and therefore, extubation was possible only a week after the operation. In the immediate postoperative period after a glossectomy, the tongue can swell to a size larger than the preoperative size. Hence, nasotracheal intubation must be maintained for several days after surgery, till swelling subsides [19].

Macroglossia caused by venous malformation can lead to several problems like dentoskeletal deformities, masticatory, and breathing difficulties. If the macroglossia continues to damage the oropharyngeal function and cause the impairment of pronunciation and sleep, it could lead to deformities. The nonunion of the jaws on one side can cause partial malunion on the other side. Considerable malunion contributes to jaw deformities like mandibular prognathism and open-bite. In this case, the patient exhibited severe mandible prognathism with anterior open-bite. However, we performed only the partial glossectomy because performing orthodontic movement was impossible in his condition, since the teeth were hypoplastic. Glossectomy was chosen as the only course of treatment to improve the patient’s chief complaint of difficulty in chewing and swallowing, due to the macroglossia. Severe mandibular prognathism, with anterior open-bite, was a contraindication to orthognathic surgery due to the severe mandibular resorption and a tendency to bleed [10, 20].

The evaluation of the tongue should include clinical, radiological, and functional assessments of speech and mastication [21]. The patient’s macroglossia compromised the airway functionality by obstruction. Thus, a partial glossectomy for the macroglossia was performed. Our results show that partial glossectomy is a reliable procedure that can result in a good surgical outcome and an improvement in the manifestations of macroglossia.

## Conclusions

In conclusion, patients with macroglossia, associated with an anterior open-bite and mandibular prognathism, require a careful selection of the method of treatment due to the high vascularity and the risk of airway obstruction. The treatment decision is based on the suitability of different approaches including sclerotherapy, electrocoagulation, and surgery. Sclerotherapy of venous malformation has been reported by numerous researchers to be effective for the treatment of venous malformations in the head and neck region. Sclerotherapy of a hemangioma is a simple, relatively innoxious, and effective treatment that places little stress on the patient and can be performed in an outpatient department. Conservative treatment is preferred, but a radical resection is required in cases where an excessively large tongue is causing functional problems and an unfavorable mandibular growth.

## Consent

Written informed consent was obtained from the patient for the publication of this report and any accompanying images.
